# Estimation of Genetic Parameters and Correlation between Yearling Ultrasound Measurements and Carcass Traits in Hanwoo Cattle

**DOI:** 10.3390/ani11051425

**Published:** 2021-05-16

**Authors:** Masoumeh Naserkheil, Deuk-Hwan Lee, Hong-Sik Kong, Jiyeon Seong, Hossein Mehrban

**Affiliations:** 1 Department of Animal Science, University College of Agriculture and Natural Resources, University of Tehran, Karaj 77871-31587, Iran; Naserkheil@ut.ac.ir; 2 Department of Animal Life and Environment Sciences, Hankyong National University, Jungang-ro 327, Anseong-si 17579, Korea; kebinkhs@hknu.ac.kr; 3 Genomic Informatics Center, Hankyong National University, Jungang-ro 327, Anseong-si 17579, Korea; s-jiyeon@hanmail.net; 4 Department of Animal Science, Shahrekord University, Shahrekord 88186-34141, Iran; hosseinmehrban@gmail.com

**Keywords:** genetic correlation, heritability, carcass traits, ultrasound, Hanwoo cattle

## Abstract

**Simple Summary:**

Knowledge of genetic parameters is essential to obtain breeding values in order to increase the response to selection and incorporate novel traits in designing a breeding program. There is a growing demand for the genetic improvement of carcass traits in the Korean beef industry. The use of yearling ultrasound measurements as indicator traits can be an efficient way to evaluate carcass traits. To date, the assessment of genetic parameters for ultrasound measurements in Hanwoo cattle is still limited. Therefore, this study was conducted to estimate the heritability, and the genetic and phenotypic correlations of yearling ultrasonic and carcass traits in Hanwoo cattle. The results revealed moderate to high heritability estimates for the traits of interest, which indicate a probable increase in the response to selection for these traits. Moreover, high and favorable genetic correlations were observed between carcass traits and their corresponding ultrasound measurements. Our findings suggest that the inclusion of yearling ultrasound data on potential replacements would be suitable as a selection tool for genetic improvement of carcass traits in Hanwoo breeding programs.

**Abstract:**

Genetic parameters have a significant role in designing a breeding program and are required to evaluate economically important traits. The objective of this study was to estimate heritability and genetic correlation between yearling ultrasound measurements, such as backfat thickness (UBFT), eye muscle area (UEMA), intramuscular fat content (UIMF), and carcass traits, such as backfat thickness (BFT), carcass weight (CW), eye muscle area (EMA), marbling score (MS) at approximately 24 months of age, as well as yearling weight (YW) in Hanwoo bulls (15,796) and steers (5682). The (co) variance components were estimated using a multi-trait animal model. Moderate to high heritability estimates were obtained and were 0.42, 0.50, 0.56, and 0.59 for CW, EMA, BFT, and MS, respectively. Heritability estimates for yearling measurements of YW, UEMA, UBFT, and UIMF were 0.31, 0.32, 0.30, and 0.19, respectively. Favorable and strong genetic correlations were observed between UIMF and MS (0.78), UBFT and BFT (0.63), and UEMA and EMA (0.65). Moreover, the estimated genetic correlation between YW and CW was high (0.84) and relatively moderate between YW and EMA (0.43). These results suggest that genetic improvement can be achieved for carcass traits when using yearling ultrasound measurements as selection criteria in ongoing Hanwoo breeding programs.

## 1. Introduction

Carcass and meat quality traits are a comprehensive term used to describe consumer perceptions of meat and are important for determining the economic profits of the beef production system. Genetic improvement of carcass traits has been a great concern in the beef cattle industry as they are expensive and difficult to measure, and can only be measured postmortem. An efficient way to evaluate carcass traits is the use of ultrasound measurements as an indicator trait in live animals. Since 1990, with the development of ultrasound technology, it has become possible to collect carcass measurements without the need to slaughter animals for recording their phenotypes [1]. Brethour [2] stated that ultrasound technology provides an opportunity to rapidly estimate genetic parameters for carcass characteristics in live animals. Furthermore, ultrasound carcass traits have the potential to increase the rate of genetic progress and reduce the required time and cost of progeny testing. Similarly, ultrasound has been shown to be an important and precise tool in the genetic evaluation and selection for intramuscular fat, subcutaneous fat thickness, muscularity, and boneless meat yield [3,4]. Based on previous studies, yearling ultrasound carcass data have been shown to have high positive genetic correlations with the corresponding carcass traits [5,6,7,8,9,10,11,12].

In recent years, beef cattle have been marketed based on carcass characteristics to meet consumer demands. In the Korean beef market, demand for high-quality animal protein is increasing, bringing meat quality into the spotlight of this industry. The Hanwoo, which is indigenous and unique to Korea, is very popular due to its rapid growth, characteristic bountiful marbling of meat, and quality attributes such as tenderness, juiciness, and good flavor [13]. The Hanwoo breeding program focuses on the estimated breeding values (EBVs) for carcass traits such as backfat thickness (BFT), carcass weight (CW), eye muscle area (EMA), and marbling score (MS) as selection criteria in efforts to increase both the quantity and quality of meat [14]. Currently, the genetic evaluation of selection candidates in Hanwoo cattle has been performed in two stages: A performance test and a progeny test for selected young bulls. The EBVs for yearling weight (YW) and MS are applied in the performance test for selecting young bulls, whereas the EBVs for BFT, CW, EMA, and MS are used in the progeny test for selecting proven bulls [15]. However, these evaluations are often not accurate or efficient, are time consuming, difficult to measure, and can only be performed when an animal reaches maturity, thereby leading to a delay in verifying the breeding results [15]. The use of genomic selection and the utilization of ultrasound-measured traits can overcome the drawbacks of these evaluation methods. Despite the economic importance of ultrasound traits, the inclusion of these traits in the selection indices for selecting young and/or proven bulls still needs to be addressed. Therefore, it is important to understand the genetic basis of the ultrasound measurements and to compute their correlations with the corresponding carcass traits, which could accelerate the progress of ongoing Hanwoo breeding programs. To this end, we estimated the genetic parameters of yearling ultrasound measurements, and the phenotypic and genetic correlations between these measurements and carcass traits using multi-trait animal models in Hanwoo cattle.

## 2. Materials and Methods

### 2.1. Animals and Phenotypic Data

Phenotypic records, including carcass traits, yearling body weight, and yearling ultrasound measurements, were selected for analyses. The dataset used in this study was provided by the Hanwoo Experiment Station, National Institute of Animal Science (NIAS), Rural Development Administration, South Korea, and included 15,796 for YW (5682 steers and 10,114 bulls), 8945 for ultrasound data (3627 steers and 5318 bulls), and 5622 Hanwoo steers, which were born between 1997 and 2017. The pedigree data were obtained from 54,284 animals. Descriptive statistics for the phenotypic dataset used for the analyses are shown in Table 1. The carcass traits were BFT, CW, EMA, and MS, which were measured according to the Korean carcass grading system in steers at approximately 24 months of age, ribbed between the 13th rib and the first lumbar vertebrae after chilling for about 24 h postmortem, according to notification No. 2014-4 of the Ministry of Agriculture, Food and Rural Affairs. MS was assessed using a categorical system of nine classes ranging from 1 (no marbling) to 9 (abundant marbling). The ultrasound traits were ultrasound backfat thickness (UBFT), ultrasound longissimus muscle area (UEMA), and ultrasound intramuscular fat content (UIMF) at the yearling age. The ultrasound traits were measured by a certified technician using a B-mode real-time ultrasound device (HS-2000, FHK Co. Ltd., Tokyo, Japan) with an 18 cm, 3.5 MHz linear probe. The animals were scanned for UEMA between the 13th rib and the first lumbar, for UBFT over the longissimus muscle at a point three-fourths the ventral length of the longissimus muscle area, and UIMF was calculated as the percentage of intramuscular fat. Analysis of the ultrasonic images with a scanning software developed by the Hanwoo Improvement Center (HIC) according to the CUP Lab at Iowa State University, USA (https://www.cuplab.com; accessed on 12 July 2007) provided the UBFT, UEMA, and UIMF phenotypes. In addition, YW for each animal was obtained from the weight (W_t_) at the termination (t) of the test (body weight at ~12 months) and the previous weight (W_t−1_) recorded at a time point (t_−1_) before (body weight at ~6 months) the termination, according to the equation described by Park et al. [15]:

$$\mathrm{YW}=\left[\left(\frac{W_{t}-W_{t_{-1}}}{t-t_{-1}}\right)\times \left(365-t_{-1}\right)\right]+W_{t_{-1}}$$

### 2.2. Statistical Analysis

The variance components and heritability of the traits were estimated through the classical Bayesian inference under a pedigree-based multi-trait animal model in the gibbsf90test software [16] as follows:

y=Xb+Zu+e

where y is the vector representing the observations for the traits of interest; b is the vector representing the fixed effects, including batch-sex-technician (108 levels), birth place (103 levels), and age of recording as a covariate for ultrasound traits; batch-sex (87 levels) and birth place (109 levels) for YW; slaughter date (274 levels), and slaughter age (days from birth to slaughter) as covariates for carcass traits; u is the vector of random genetic additive effects; e is the vector of random residual effects; and X and Z are incidence matrices related to the fixed and random genetic additive effects, respectively. 

In this study, it was assumed that random effects of u and e followed the multivariate normal distribution with the mean of zero and Var (u) = G⊗A and Var(e) = R⊗I, in which A, G, R, and I are the matrices of the numerator relationship, (co) variances of additive genetic effects, residual, and identity, respectively, as well as ⊗ is the Kronecker product. Moreover, prior distributions of G and R were defined as a flat prior according to the software default. In addition, genetic and phenotypic correlations and their corresponding standard deviations were obtained from multi-trait analyses.

The Markov Chain Monte Carlo (MCMC) method was used to estimate variance components and heritability with 50,000 iterations as burn-in with a thinning interval of 50 in 550,000 cycles. In addition to these criteria, the convergence of the chain was evaluated by visual inspection of the trace plots. After verification of the convergence of the Gibbs chain, the variance components and correlations were estimated as the posterior means of the corresponding sampled values. 

## 3. Results and Discussion

### 3.1. Descriptive Statistics and Heritability Estimates

This study was conducted to estimate the genetic parameters of yearling ultrasound measurements and their correlations with carcass traits in Hanwoo beef cattle. The descriptive statistics for the carcass, yearling body weight, and ultrasound traits are shown in Table 1. The mean values of the studied traits ranged from 2.57 to 370.48, with the standard deviation (SD) between 1.05 and 44.07. A considerably higher phenotypic coefficient of variation (CV) was observed for UBFT, UIMF, BFT, and MS (28.55 to 55.57%), whereas the variations were relatively low (11.00 to 14.59%) for the other traits. Table 2 shows the estimates of the variance components and heritability for the studied traits. The estimated heritability of the eight analyzed traits were moderate to high, ranging from 0.19 to 0.59 (Table 2), indicating considerable additive genetic variation in most of the traits. The estimates of posterior heritability for the carcass traits were 0.56 (BFT), 0.42 (CW), 0.50 (EMA), and 0.59 (MS), which were comparable to those previously reported in Hanwoo cattle [15,17,18,19,20,21,22]. Our heritability estimate of 0.31 for YW was similar to the estimates of 0.26 and 0.30 obtained by Mehrban et al. [21] and Park et al. [15], respectively, but was higher than the estimate of 0.18 obtained by Choi et al. [19] in Hanwoo beef cattle. In addition, the heritability estimated for YW was lower than the results reported by Moser et al. [5] and Johnson et al. [23] for Brangus (0.40 and 0.44, respectively), by Kemp et al. [8] for Angus (0.55), and by Elzo et al. [24] for multibreed Angus-Brahman (0.54) cattle. However, it was similar to those reported by Zuin et al. [25] and Yokoo et al. [26] for Nellore (0.29 and 0.32, respectively), and by Koots et al. [27] for Angus cattle (0.33). Carcass traits were found to be moderately to strongly heritable, indicating that there is potential to improve these traits through genetic selection. The estimated posterior heritabilities for the ultrasound carcass traits UIMF, UEMA, and UBFT were 0.19, 0.32, and 0.30, respectively. In the present study, the heritability estimate for UEMA was comparable to previously reported values of 0.17 [10], 0.23 [28], 0.31 [29], and 0.16 [18] in Hanwoo cattle. In other breeds, heritability estimates of UEMA, similar to our results, were obtained in multi-breed Angus-Brahman [12], Angus [6,8,30], Brangus [5,31], Simmental [9], and Nellore cattle [11,32,33]. For UBFT, the moderate estimate of heritability in this study was within the range of reported estimates of 0.30 for Angus [34], 0.26 for Brangus [31], 0.22 for multi-breed Angus–Brahman [24], and 0.31 for Canadian crossbred cattle [35]. However, our heritability estimate for UBFT was lower than the values estimated by Kemp et al. [8] (0.39) and Shepard et al. [36] (0.56) in Angus, Reverter et al. [6] (0.51) in Hereford and Angus, Crews et al. [9] (0.53) in Simmental, and Yokoo et al. [11] (0.42) and Bonin et al. [33] (0.44) in Nellore cattle. Similarly, Lee and Kim [10] and Hwang et al. [18] reported heritability estimates of 0.52 and 0.43, respectively, for UBFT in Hanwoo steers, which was higher than the present findings. Our heritability estimate for UIMF was comparable to the estimate (0.16) of Stelzleni et al. [31] for Brangus yearling bulls and heifers and the estimate of 0.25 by Kelly et al. [30] from a combined dataset of bulls, steers, and heifers in Angus, whereas it was lower than the estimates obtained in the other literature. For instance, Lee and Kim [10] and Hwang et al. [18] showed that the estimated heritability for UIMF was 0.55 and 0.33, respectively, in Hanwoo steers. Additionally, Bertrand et al. [37] and Reverter et al. [6] reported a heritability of 0.41 and 0.43, respectively, for intramuscular fat percent measured using ultrasound. In other breeds, the UIMF values of 0.47 for yearling Simmental bulls [9], 0.29 for Red Angus [38], 0.43 and 0.30 for multi-breed Angus–Brahman [12,24], and 0.37 for Canadian crossbred cattle [35] were higher than those obtained from our results. Overall, several factors may be responsible for the discrepancy in heritability estimates between the present and previous studies, such as differences in the sample size, breed, age of measurement of ultrasound data, completeness of the pedigree, and statistical models used for (co) variance estimation. 

### 3.2. Genetic and Phenotypic Correlations 

The genetic and phenotypic correlations between yearling body weight, ultrasound, and carcass traits are presented in Table 3. 

The estimated genetic correlations among the carcass traits were of low (close to zero) to moderate magnitude, ranging from 0.03 ± 0.07 to 0.56 ± 0.05. As shown in Table 3, the strong, positive genetic correlation between CW and EMA (0.56 ± 0.05) obtained in our study is in agreement with the previously reported estimates in Hanwoo [19,20], Brahman [39,40], Hereford, and Simmental cattle [41]. This result indicates that the selection to increase the carcass weight will result in animals with greater EMA. Our results showed that the estimated genetic correlation between EMA and MS was relatively moderate and positive (0.35 ± 0.06), similar to those obtained from earlier studies [19,39,40], but was substantially lower than the estimate of 0.65 reported by Hwang et al. [18] in Hanwoo cattle. However, a negative and relatively low genetic correlation was observed between BFT and EMA (−0.25 ± 0.07), whereas the genetic correlation of BFT with the two other carcass traits (CW and MS) was negligible and close to zero, indicating that the selection for one trait may have a trivial effect on the other traits. Additionally, the phenotypic correlations among the carcass traits, which ranged from near zero (0.01 ± 0.02) to moderate (0.57 ± 0.01), indicated a similar trend to those of the genetic correlations.

Analysis of the estimated correlations among YW and carcass traits revealed that the highest genetic and phenotypic correlation occurred between YW and CW, at 0.84 ± 0.02 and 0.78 ± 0.01, respectively. Consequently, the selection for increased YW will result in favorable correlated responses in CW. Similar results have been reported by Choi et al. [19] and Elzo et al. [12] who estimated genetic correlations of 0.77 and 0.63 between YW and CW, respectively. Moreover, the genetic correlation between YW and EMA was relatively moderate and positive (0.43 ± 0.06), whereas the genetic correlation of YW with the two other carcass traits, BFT and MS was nearly zero, suggesting that the selection for YW has no effect on BFT and MS traits. These estimates were similar to the those obtained by Elzo et al. [12] for the correlation of YW with EMA and MS, at 0.36 and 0.03, respectively, except for the correlation with BFT, which had a higher magnitude of 0.35. 

The estimates for the genetic correlation between yearling ultrasound and carcass traits were very low or near zero to strong and positive, and varied from −0.01 ± 0.07 to 0.78 ± 0.04. Concerning the correlations between corresponding ultrasound measures and carcass traits, the highest genetic correlation was observed between UIMF and MS (0.78 ± 0.04), followed by the correlation between UEMA and EMA (0.65 ± 0.05), and between UBFT and BFT (0.63 ± 0.05). The strong and positive genetic correlations between carcass traits and their corresponding ultrasound measurements reaffirm the usefulness of ultrasound measurements as indicators of carcass traits for informing genetic evaluation programs of beef cattle. Similar estimates of the genetic correlation between UIMF and MS were found by Wilson et al. [42] for Angus (0.77), by Devitt and Wilton [43] and Crews et al. [9] for Simmental (0.80 and 0.74, respectively), by Elzo et al. [12] for multi-breed Angus-Brahman (0.56), and by Su et al. [41] for Hereford (0.54) and Simmental cattle (0.73). In previous studies, the genetic correlation of UEMA with EMA ranged from 0.29 to 0.94 [5,6,9,12,41,43,44]. In addition, the estimated genetic correlation between UBFT and BFT in the present study was comparable to the previously reported values of 0.88 for Simmental [43], 0.79 and 0.83 for Simmental bulls and heifers [9], 0.82 and 0.96 for Angus bulls and heifers [6], 0.69 for multi-breed Angus-Brahman [12], and 0.74 for Hereford cattle [41]. Estimated genetic correlations between UIMF and other traits were 0.04 (UEMA), 0.46 (UBFT), 0.11 (BFT), 0.14 (CW), 0.28 (EMA), and −0.05 (YW). The estimated genetic correlations between UEMA and with other traits in our study were 0.16 (UBFT), −0.09 (BFT), 0.40 (CW), −0.07 (MS), and 0.41 (YW). The estimated genetic correlations between UBFT and other traits were −0.01 (CW), −0.12 (EMA), 0.14 (MS), and 0.15 (YW). The moderately positive genetic correlations of UEMA with CW (0.40 ± 0.06) and YW (0.41 ± 0.05) in this study were comparable to those previously reported in the literature for various breeds of cattle [5,8,11,12,31,45]. Similarly, there was a reasonable agreement between estimates of genetic correlations between UBFT and UIMF (0.46 ± 0.06) from our study with those obtained in previous studies (0.39 [46], 0.36 [31], 0.36 [12]). However, our results were considerably higher than the estimate of 0.02 reported by Kemp et al. [8] in Angus cattle. The UBFT was very low (0.16 ± 0.07) and positively correlated with UEMA, and was lower than the results reported by Arnold et al. [46] (0.48) and Evans et al. [47] (0.38) but similar to those reported by Johnson et al. [23] (0.12) and Moser et al. [5] (0.13). Additionally, several recent studies have reported moderate to strong genetic correlations between carcass traits and growth and ultrasound traits in sheep population [48,49,50,51]. For instance, Massender et al. [51] reported that ultrasound measured eye muscle depth and fat depth were found to be moderately to strongly positively correlated with hot carcass weight (0.33 to 0.71) and fat depth (0.38 to 0.74), respectively in the Canadian sheep population. 

The phenotypic correlations between ultrasound and carcass traits generally showed the same tendency as the genetic correlations but were of lower magnitude, ranging from almost zero to moderate. The largest phenotypic correlations were observed between UEMA and YW (0.51 ± 0.01), UEMA and EMA (0.48 ± 0.01), UEMA and CW (0.42 ± 0.01), UIMF and MS (0.42 ± 0.01), and between UBFT and BFT (0.40 ± 0.01). The phenotypic correlations in this study were within the range of values estimated previously in multibreed Angus-Brahman [12], Angus [6,8], Brangus [5,31], Nellore [11], and Canadian crossbred cattle [35]. The moderate to high heritability of ultrasound and carcass traits, as well as the positive and rather high genetic correlation between carcass and ultrasound measurements obtained in the present study indicates that the advantages of utilizing ultrasound information would be particularly important to improve the genetic evaluation and selection of cattle for carcass traits. 

## 4. Conclusions

The results of this study demonstrate that carcass and yearling ultrasound traits are heritable, and the sufficient additive genetic variability observed for these traits indicates that the selection for these traits can be effective. In addition, high and favorable genetic correlations between carcass traits and their corresponding ultrasound measurements suggest that the inclusion of yearling ultrasound data as a selection tool can provide a good opportunity for genetic improvement of carcass traits in Hanwoo breeding programs. Therefore, knowledge of the genetic parameters of the ultrasound-measured traits can be effective to obtain more accurate breeding values to increase the response to the selection and reduce the expense and generation interval, which in turn could lead to considerable profitability in the Hanwoo beef industry.

## Figures and Tables

**Table 1:** Descriptive statistics for yearling ultrasound data and carcass traits in Hanwoo cattle.

Trait (Unit)	Number of Records	Mean (SE)	Min.	Max.	SD	CV (%)
UIMF (%)	8943	2.57 (0.02)	0.10	13.40	1.43	55.57
UEMA (cm^2^)	8945	54.11 (0.08)	24.20	88.70	7.90	14.59
UBFT (mm)	8945	3.68 (0.01)	1.10	9.10	1.05	28.55
BFT (mm)	5622	9.92 (0.05)	1.00	35.00	3.95	39.83
CW (kg)	5619	370.48 (0.57)	213.00	562.00	42.80	11.55
EMA (cm^2^)	5617	81.62 (0.12)	50.00	121.00	8.98	11.00
MS (1–9)	5622	3.53 (0.02)	1.00	9.00	1.64	46.50
YW (kg)	15,796	357.13 (0.35)	190.49	547.65	44.07	12.34

UIMF: Ultrasound intramuscular fat content; UEMA: Ultrasound longissimus muscle area; UBFT: Ultrasound backfat thickness; BFT: Backfat thickness; CW: Carcass weight; EMA: Eye muscle area; MS: Marbling score; YW: Yearling weight; SE: Standard error; SD: Standard deviation; CV: Coefficient of variation.

**Table 2:** Heritability and variance component estimated from pedigree and phenotypic information using the multi-trait model in the Hanwoo cattle.

Trait	$\sigma_{a}^{2}$	$\sigma_{e}^{2}$	$\sigma_{p}^{2}$	$h^{2}$
UIMF	0.23 (0.03)	0.98 (0.03)	1.21 (0.02)	0.19 (0.02)
UEMA	9.98 (0.97)	20.72 (0.76)	30.70 (0.51)	0.32 (0.03)
UBFT	0.21 (0.02)	0.48 (0.02)	0.69 (0.01)	0.30 (0.03)
BFT	7.15 (0.68)	5.59 (0.53)	12.74 (0.29)	0.56 (0.05)
CW	596.32 (61.58)	828.31 (48.54)	1424.63 (32.01)	0.42 (0.04)
EMA	34.60 (3.58)	34.40 (2.81)	68.99 (1.63)	0.50 (0.04)
MS	1.43 (0.13)	0.97 (0.10)	2.40 (0.05)	0.59 (0.05)
YW	354.09 (32.86)	792.65 (24.94)	1146.74 (15.93)	0.31 (0.03)

UIMF: Ultrasound intramuscular fat content; UEMA: Ultrasound longissimus muscle area; UBFT: Ultrasound backfat thickness; BFT: Backfat thickness; CW: Carcass weight; EMA: Eye muscle area; MS: Marbling score; YW: Yearling weight; $\sigma_{a}^{2}$: Additive genetic variance; $\sigma_{e}^{2}$: Error variance; $\sigma_{p}^{2}$ Phenotypic variance; $h^{2}$: Heritability. Numbers in parentheses are the standard deviations of posterior densities.

**Table 3:** Estimates of genetic (above diagonal) and phenotypic (below diagonal) correlations among the yearling ultrasound measurements and carcass traits in Hanwoo cattle.

Trait	UIMF	UEMA	UBFT	BFT	CW	EMA	MS	YW
UIMF		0.04 (0.07)	0.46 (0.06)	0.11 (0.08)	0.14 (0.07)	0.28 (0.07)	0.78 (0.04)	−0.05 (0.07)
UEMA	0.05 (0.01)		0.16 (0.07)	−0.09 (0.07)	0.40 (0.06)	0.65 (0.05)	−0.07 (0.08)	0.41 (0.05)
UBFT	0.15 (0.01)	0.22 (0.01)		0.63 (0.05)	−0.01 (0.07)	−0.12 (0.07)	0.14 (0.07)	0.15 (0.07)
BFT	0.07 (0.01)	0.08 (0.02)	0.40 (0.01)		0.07 (0.07)	−0.25 (0.07)	0.03 (0.07)	−0.05 (0.07)
CW	0.04 (0.01)	0.42 (0.01)	0.15 (0.01)	0.27 (0.02)		0.56 (0.05)	0.17 (0.07)	0.84 (0.02)
EMA	0.09 (0.01)	0.48 (0.01)	0.05 (0.01)	0.01 (0.02)	0.57 (0.01)		0.35 (0.06)	0.43 (0.06)
MS	0.42 (0.01)	0.01 (0.02)	0.08 (0.02)	0.08 (0.02)	0.12 (0.02)	0.24 (0.02)		0.03 (0.07)
YW	0.05 (0.01)	0.51 (0.01)	0.23 (0.01)	0.18 (0.02)	0.78 (0.01)	0.40 (0.01)	0.06 (0.02)	

UIMF: Ultrasound intramuscular fat content; UEMA: Ultrasound longissimus muscle area; UBFT: Ultrasound backfat thickness; BFT: Backfat thickness; CW: Carcass weight; EMA: Eye muscle area; MS: Marbling score; YW: Yearling weight. Numbers in parentheses are the standard deviations of posterior densities.

## Data Availability

The data presented in this study are available at http://www.limc.co.kr and http://www.ekape.or.kr.
